# Genomic Diversity in Two Related Plant Species with and without Sex Chromosomes - *Silene latifolia* and *S. vulgaris*

**DOI:** 10.1371/journal.pone.0031898

**Published:** 2012-02-29

**Authors:** Radim Cegan, Boris Vyskot, Eduard Kejnovsky, Zdenek Kubat, Hana Blavet, Jan Šafář, Jaroslav Doležel, Nicolas Blavet, Roman Hobza

**Affiliations:** 1 Department of Plant Developmental Genetics, Institute of Biophysics, Academy of Sciences of the Czech Republic, v.v.i., Brno, Czech Republic; 2 Department of Plant Biology, Faculty of Agronomy, Mendel University in Brno, Brno, Czech Republic; 3 Centre of the Region Haná for Biotechnological and Agricultural Research, Institute of Experimental Botany, Olomouc, Czech Republic; 4 Institute of Integrative Biology, Plant Ecological Genetics, ETH Zurich, Zurich, Switzerland; Oregon State University, United States of America

## Abstract

**Background:**

Genome size evolution is a complex process influenced by polyploidization, satellite DNA accumulation, and expansion of retroelements. How this process could be affected by different reproductive strategies is still poorly understood.

**Methodology/Principal Findings:**

We analyzed differences in the number and distribution of major repetitive DNA elements in two closely related species, *Silene latifolia* and *S. vulgaris*. Both species are diploid and possess the same chromosome number (2n = 24), but differ in their genome size and mode of reproduction. The dioecious *S. latifolia* (1C = 2.70 pg DNA) possesses sex chromosomes and its genome is 2.5× larger than that of the gynodioecious *S. vulgaris* (1C = 1.13 pg DNA), which does not possess sex chromosomes. We discovered that the genome of *S. latifolia* is larger mainly due to the expansion of *Ogre* retrotransposons. Surprisingly, the centromeric STAR-C and TR1 tandem repeats were found to be more abundant in *S. vulgaris*, the species with the smaller genome. We further examined the distribution of major repetitive sequences in related species in the *Caryophyllaceae* family. The results of FISH (fluorescence *in situ* hybridization) on mitotic chromosomes with the *Retand* element indicate that large rearrangements occurred during the evolution of the *Caryophyllaceae* family.

**Conclusions/Significance:**

Our data demonstrate that the evolution of genome size in the genus *Silene* is accompanied by the expansion of different repetitive elements with specific patterns in the dioecious species possessing the sex chromosomes.

## Introduction

Angiosperm species display large variability in genome size, ranging from 1C = 0.0648 pg in the carnivorous species *Genlisea margarethae* [1] to 1C = 152.23 pg in the monocot *Paris japonica* [2]. Even genomes of closely related species vary significantly in size and structure and this phenomenon was termed C-value paradox [3]. Such patterns are especially obvious in plants where genome dynamism is higher than in animals. During the past few decades the components contributing to differences in genome size were characterized. However, the reason for the variation remains unclear and the term C-value enigma was introduced to reflect this [4].

The major mechanisms of genome size increase are polyploidization [5] and the expansion of different repetitive elements [6], [7], [8]. In this work, we focus on the latter mechanism. Transposable elements (TEs) are widespread in all eukaryotes and may have a significant impact on genome size dynamics [9]. They have been classified in two separate classes based on their mode of transposition [10]. The first class contains elements transposing via an RNA intermediate. This class is further divided based on terminal sequences and includes long-terminal repeat (LTR) retrotransposons (*Ty1/copia*-like and *Ty3/gypsy-like*), transposons terminating at the 3^′^ end with a poly(A) tract (LINEs - long interspersed nuclear elements, SINEs - short interspersed nuclear elements), and other orders like DIRS (*Dictyostelium* intermediate repeat sequence) and PLE (Penelope-like elements) retrotransposons [10]. The proliferation of LTR-retrotransposons has been recognized as one of the key mechanisms accounting for genome expansion in plants [11]. The second class of TEs is transposed via a DNA intermediate. It has been grouped into several superfamilies such as *hAT*, *CACTA*, *Mutator*-like or *Tc1/Mariner*.

Another important mechanism of plant genome expansion is the proliferation of satellite DNA [12], [13]. In addition to many conserved repeated units including rDNA and telomeric and centromeric repeats, there is a large variation in other types of tandemly organized DNA that form plant genomes. Tandem repeats are arranged in microsatellites (2–5 bp units), minisatellites (up to 100 bp per unit), and larger satellites.

In this study, we investigate genome size evolution by comparing two closely related species from the *Caryophyllaceae* family, *Silene latifolia* and *S. vulgaris*. Both species belong to the genus *Silene* (subgenus *Behenantha*). *S. latifolia* Poiret (syn. *Melandrium album* Garcke, syn. *Melandrium pratense* Roehl.) is a strictly dioecious species with separate male and female individuals determined by the sex chromosomes. *S. vulgaris* is a gynodioecious species with female and hermaphroditic individuals. In this species the sex is controlled by interactions of nuclear (autosomal) factors and mitochondrial genes in the process called cytoplasmic male sterility [14].

The structure and evolution of the entire *S. latifolia* genome has been studied more extensively, mainly in the context of sex chromosome evolution [15], [16]. Many tandem repeats in *S. latifolia* are localized in the vicinity of telomeres [17], [18], [19] and/or specifically linked to the sex chromosomes [20], [21]. A comprehensive study of microsatellite distribution in *S. latifolia* [22] relied on fluorescence *in situ* hybridization (FISH) and showed that specific sequence repeats are overrepresented on the Y chromosome. It has also been demonstrated that chloroplast DNA had been transferred and accumulated on *S. latifolia* sex chromosomes during their evolution [23]. The authors identified chloroplast sequences in Y chromosome specific genomic library. Moreover, FISH results with BAC clone containing part of chloroplast genome revealed strong signal intensity on the Y chromosome. Matsunaga *et al.* [24], Obara *et al.* [25], and Kejnovsky *et al.* [26] studied the structure and evolution of specific retrotransposons in *S. latifolia*. Pritham *et al.* [27] isolated the first transcriptionally active DNA transposon linked to the Y chromosome. A systematic study of repetitive DNA in *S. latifolia* showed that *Copia* retroelements are probably the most abundant DNA element on the Y chromosome [28]. The first active MITE (miniature inverted-repeat transposable element) elements in *S. latifolia* were described by Bergero *et al.* [29].

These studies reflect the increasing data about the structure of *S. latifolia* genome mainly with a special focus on sex chromosome evolution; however, there has not been a comparison among closely related species to distinguish which types of elements played a role in genome size evolution generally, and which elements played a role in sex chromosome evolution specifically. For *S. vulgaris* there is almost no data about the structure and evolution of major repetitive elements in its genome. There exists only fragmented information about the differences between *S. latifolia* and *S. vulgaris* genomes, which comes from studies focused on the characterization of some gene regions [30], [31].

Here we address the following questions: What makes the *S. latifolia* genome larger in comparison with *S. vulgaris*? Is the smaller genome of *S. vulgaris a priori* with a lower copy number of individual repetitive elements? Do all repetitive elements amplify in a bigger genome? Is there a space for the large duplication events that might have formed *S. latifolia* genome? Is *S. latifolia* genome larger just based on expansion of repetitive DNA compared to *S. vulgaris*? Which DNA elements play a specific role in formation of the sex chromosomes?

## Results

### Differences in the abundance of individual repetitive elements in *S. latifolia* and *S. vulgaris* genome

We constructed a short-insert DNA library of *S. vulgaris*. The library contained 7,720 clones with an average insert size of 603 bp representing an equivalent of about 0.42% of *S. vulgaris* genome (1C = 1.13 pg DNA = 1102.50 Mbp [32]). In order to isolate clones containing repetitive elements, TEs and tandem repeats, we amplified the conservative domains of *gypsy* and *copia-like* reverse transcriptases, LINE endonuclease, Au SINE, *CACTA*, *Mariner* and *Mutator* transposase, STAR-C, TR1 and X.43.1 tandem repeats and used them as probes for a hybridization-based library screening. We applied a complex genomic DNA probe in parallel to identify other repetitive elements. After screening the library, all positively hybridizing clones were sequenced and the presence of specific repeats was confirmed. The number of clones containing each of the individual repeats is summarized in Table S1. In order to make our data comparable with those obtained by Cermak *et al.* [28] for *S. latifolia*, we calculated the relative number of clones containing specific elements and the percentage of the genome formed by this element (Table 1).

**Table 1:** The total number of clones from the *S. vulgaris* short insert library containing respective elements and the proportion of total screened clones in comparison with the *S. latifolia* [28].

Type of element					
	*Silene vulgaris*		Silene latifolia *		
	number of sequences per genome	% of genome	number of sequences per genome	% of genome	Ratio %SV/%SL
**Retroelements**	29540	22.9%	**72500**	**36.4%**	**0.63**
**LTR**	29064	22.8%	**69687**	**36.1%**	**0.63**
**Gypsy**	**13112**	**12.7%**	**55937**	**32.5%**	**0.39**
Athila	714	0.5%	10000	2.7%	0.18
Ogre	0	0	25000	22.7%	0
Peabody	1904	1.1%	11250	2.8%	0.39
Retand	11190	11.2%	9687	4%	2.8
**Copia**	15952	10.1%	**13750**	**3.6%**	**2.81**
**Non - LTR**	**476**	**0.10%**	**2813**	**0.3%**	**0.33**
SINE	0	0	0	0	0
LINE	476	0.10%	2813	0.3%	0.33
**DNA transposons**	**1428**	**0.6%**	**938**	**0.2%**	**3**
**Tandem repeats**	**42141**	**0.3%**	**42814**	**0.46%**	**0.65**
Tandem repeat STAR	28095	0.1%	9063	0.02%	5
Tandem repeat X.43.1	4523	0.12%	31563	0.37%	0.32
TR1	9523	0.06%	2188	0.01%	6
**All repetitive elements**	**73109**	**23.4%**	**116252**	**36.8%**	**0.64**

Ratios % SV/% SL below 1.0 indicate elements that are more expanded in *S. latifolia* genome, and ratios % SV/% SL higher than 1.0 show elements that are more expanded in *S. vulgaris*.

As in the *S. latifolia* genome, retroelements represented the most repetitive fraction of *S. vulgaris* genome. However, the genome fraction comprised by retroelements was 1.6× higher in *S. latifolia* than in *S. vulgaris*, indicating that retroelements have played a dominant role in the expansion of the *S. latifolia* genome. The contribution of individual retroelements differed significantly. The most extreme values were observed for the *Ogre* retroelement group which is present in 25 thousands of copies in the *S. latifolia* genome (∼23% of its genome). Based on shotgun genomic library screening this element is absent in *S. vulgaris* genome. On the other hand, the *Retand* retroelement represented ∼11% of *S. vulgaris* genome compared to only 4% in *S. latifolia*. The *Athila* and *Peabody* retroelements were also more abundant in *S. latifolia*. The *Copia* type retrotransposons represented ∼10% of *S. vulgaris* genome compared to ∼4% in *S. latifolia*. Non-LTR (SINE, LINE) retrotransposons appear to have played only a minor role in genome size divergence between the species. DNA transposons are about 2× more common in *S. vulgaris* compared to *S. latifolia*, but as with non-LTR retrotransposons they represent only a minor fraction of the genome in both species. Surprisingly, centromeric tandem repeats STAR-C and TR1 are almost 5× more abundant in *S. vulgaris* than in *S. latifolia*. The opposite is true of the subtelomeric tandem repeat X.43.1, which is 3× more abundant in the *S. latifolia* genome. Although the percentage of these elements in the genome seems to be rather low in both species, this percentage does not take into account tandem (i.e. the number of repeat units) in individual sequenced clones (one clone was calculated as one hit) since such data is not yet available for *S. latifolia*. Taken together, the majority of the TEs were more abundant in the *S. latifolia* genome. On the other hand, some TEs like *Retand* or STAR-C centromeric tandem repeat formed a larger proportion of the *S. vulgaris* genome. Overall, the repetitive elements we identified in the *S. vulgaris* and *S. latifolia* genomes represented ∼23% and ∼36% of entire genome, respectively.

We used Southern blot hybridization to estimate the abundance of *Ogre*, *Retand*, and *Copia* retroelements in both the *S. latifolia* and *S. vulgaris* genomes. We used the reverse transcriptase domain for *Ogre* and *Copia* retroelements, and the pol domain for *Retand* as a probes. This hybridization data clearly showed that the *Ogre* retroelement was missing in the *S. vulgaris* genome (Figure S1). The *Retand* and *Copia* retroelements are similarly abundant in both genomes. Except for the *Ogre* retroelement, the hybridization patterns of all the studied elements are similar in both species. Comparisons of hybridization signals between genomic DNA restricted by the methylation sensitive *HpaII* enzyme and its isoschizomer *MspI* showed that these three analyzed elements are partially methylated in both genomes.

### Chromosomal distribution and evolutionary divergence of repetitive DNA

**Figure 1:**
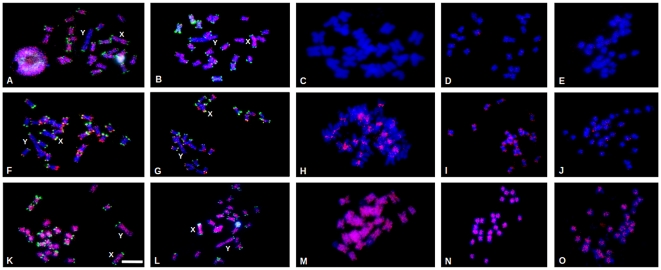
Chromosomal distribution of the reverse transcriptases of *Ogre*, *Retand* and *Copia* retroelements in five species from the *Caryophyllaceae* family as revealed by FISH. Mitotic metaphase chromosomes of *S. latifolia* (A, F, K), *S. dioica* (B, G, L), *S. chalcedonica* (C, H, M), *S. vulgaris* (D, I, N) and *Dianthus caryophyllus* (E, J, O) were counterstained with DAPI (blue). The probes for each element (*Ogre* – reverse transcriptase (A–E), *Retand* – reverse transcriptase (F–J) and *Copia* – reverse transcriptase (K–O)) were labeled with Cy3-conjugated nucleotides (red). The probe for X.43.1. subtelomeric repeat (A, B, F, G, K, L) was labeled with Spectrum Green (green). The X and Y chromosomes are indicated, bar indicates 10 µm.

To show patterns of individual repetitive elements on chromosomes of *S. latifolia*, *S. vulgaris* and some other closely related species, we employed FISH with the most abundant repeats (Figure 1). The probes used for FISH spanned the conservative parts of individual repetitive elements identified by genomic library screening. Based on abundance we selected *Ogre* and *Retand* elements (*gypsy* retrotransposons), *Copia* retroelements, and tandem repeats STAR-C and TR1 for further experiments. The 25S rDNA and X.43.1 repeats were used as internal controls. We selected five species from the *Caryophyllaceae* family (genus *Silene*: *S. latifolia*, *S. dioica*, *S. vulgaris*; genus *Lychnis*: *L. chalcedonica*, genus *Dianthus*: *D. caryophyllus*) for FISH experiments. Our aim in this set of experiments was to compare the distribution and organization of these elements in order to make inferences about both genome and sex chromosome evolution.

The *Ogre* retroelement, the most abundant repetitive sequence in *S. latifolia*, showed, in agreement with the previously published data [28], [33], a random distribution over all the chromosomes, but almost no signal on the Y chromosome. We observed a similar pattern in a closely related dioecious plant *S. dioica*. Interestingly, we found no evidence of the *Ogre* retroelement in *L. chalcedonica*, *S. vulgaris* and *D. caryophyllus*. The *Retand* retroelement, which was linked to the subtelomeric region in *S. latifolia* [26], was localized to the terminal chromosomal regions of all the chromosomes in *S. latifolia*, *S. dioica* as well as in *S. vulgaris* in agreement with a previous report [26]. In *D. caryophyllus* only several major signals were observed. Surprisingly, completely different pattern of distribution was found in *L. chalcedonica*; almost all the chromosomes possessed strong centromeric signals with no hybridization signals in the terminal parts of the chromosomes. *Copia* retrotransposons showed a relatively evenly spread distribution in all chromosomes (with the exception of a missing signal in the subtelomeric regions) in all studied species.

The tandem repeat STAR-C was localized at the centromeres in all studied species (Figure S2). Only *D. caryophyllus* had some chromosomes without any signal. In order to show differences in abundance of STAR-C in *S. latifolia* and *S. vulgaris* (∼5× more abundant in the *S. vulgaris* genome compared to *S. latifolia* (Table 1)), we took images of metaphase chromosomes after FISH hybridization with STAR-C probe with different exposure times (Figure S3). This “quantitative” FISH of STAR-C confirmed that this repeat was more abundant in *S. vulgaris*. The TR1 tandem repeat showed subtelomeric organization corresponding to rDNA regions in *S. latifolia*, *S. dioica*, *S. vulgaris*, and *L. chalcedonica*. Co-localization of TR1 with rDNA loci was previously demonstrated by Cermak *et al.* in *S. latifolia* [28]. The only exception was *D. caryophyllus*, where we did not find any TR1 hybridization. The X.43.1 tandem repeat was subtelomerically organized in the *S. latifolia*, *S. dioica* and *S. vulgaris* genomes. In *L. chalcedonica* X.43.1 was localized only at several dominant subtelomeric and minor centromeric loci. In *D. caryophyllus* X.43.1 was completely missing (Figure S2).

### Comparative analysis of complete *Retand* retrotransposons from *S. latifolia* and *S. vulgaris*

**Figure 2:**
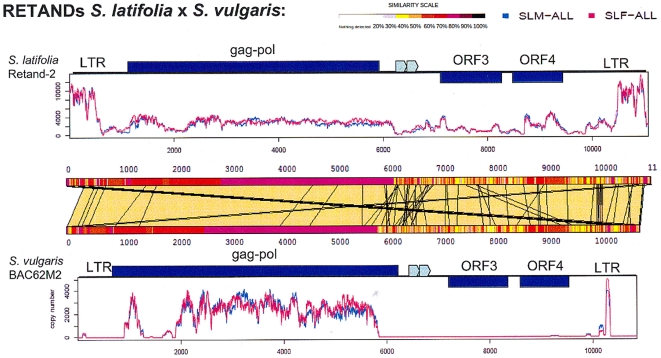
Comparison of *S. latifolia* (Retand-2) and *S. vulgaris* (BAC62M2) *Retand* retrotransposons. *Retand* elements are represented on the X axis with indicated length in bp. The plots represent genomic copy numbers of individual insert regions calculated from numbers of similarity hits to the 454 read databases [35] of both male (SLM-all, blue) and female (SLF-all, red) *S. latifolia* individuals. The central panel displays a comparison of similarity of individual retroelements. Specific domains of retroelements are indicated.

We compared the sequence of *Retand*-2 [26] of *S. latifolia* with the *Retand* element from BAC clone 62M2 from *S. vulgaris* containing *Sv4* gene previously characterized in this species [31], [34]. We measured coverage of both elements with 454 sequencing reads [35] obtained from *S. latifolia* male and female genomes (Figure 2). *Retand*-2 was homogenously represented in both male and female genomic 454 reads with higher abundance of LTRs suggesting the presence of solo LTRs in *S. latifolia* genome (upper part of Figure 2). The *Retand* element of *S. vulgaris* origin exhibited the highest degree of identity with *S. latifolia* 454 reads in the gag-pol region (lower part of Figure 2). It was in accordance with alignment of both *Retand* elements showing their highest similarity in the central region (middle part of Figure 2).

### Analysis of microsatellite sequences in corresponding genomic loci in *S. latifolia* and *S. vulgaris*

To analyze the extent and pattern of distribution of different microsatellite sequences, we counted all di-, tri- and tetra- microsatellites (Table S2) in two gene regions linked to the sex chromosomes in *S. latifolia* (*Sl4X/Y*, *SlAP3X/Y*) and the corresponding autosomal region in *S. vulgaris*. BAC clones for all these loci were previously identified and characterized by Marais *et al.* [30] and Cegan *et al.* [31]. Separately we summarized data on dinucleotides CA, GA, GC and trinucleotides CAG, CAG, GAA, TAA, which were shown to be accumulated on Y chromosome of *S. latifolia* [22]. However, we did not find a specific accumulation of any microsatellite type in the Y linked sequences compared to the X and autosomal loci. Longer stretches of microsatellites (>18 bp) were almost completely missing in the analyzed sequences (Table S3). In the *SlAP3Y* gene linked genomic locus, the largest dinucleotide array (AC) was composed of 50 bp, trinucleotide of 48 bp (GAA), and tetranucleotide of 42 bp (ATAC). In the *Sl4Y* gene linked genomic locus, the largest dinucleotide array (TA) was composed of 54 bp, trinucleotide of 96 bp (TTA), and tetranucleotide of 16 bp (ACAA). Taken together, the abundance of microsatellites was similar in BAC clones from homologous regions in *S. latifolia* and *S. vulgaris*, without any accumulation on the sex chromosomes. Some of the microsatellites that are known to accumulate on the Y chromosome in *S. latifolia* [22] formed the longest arrays in the studied BAC clones (AC, TA, GAA).

## Discussion

### What makes the *S. latifolia* genome larger than the *S. vulgaris* genome?

It is known that retrotransposons play a dominant role in genome size evolution in both angiosperm and in gymnosperm species [36]. To get a profile of the most abundant DNA sequences in *S. vulgaris*, we employed two different strategies. First, we amplified the conservative parts of different retrotransposons and other repeats found and characterized in closely related species and used amplicons as the probes for hybridization with the random genomic library. To identify other abundant elements specific to the *S. vulgaris* genome we screened the genomic DNA in parallel. We avoid the next generation sequencing (NGS) methods to make our data comparable to Cermak *et al.* [28] who used identical methodical approach in *S. latifolia*. Moreover, a recent paper comparing 454 based estimation of repetitive DNA proportion in *S. latifolia* with data by Cermak *et al.* [28] reveals similar results and conclusions. Even though NGS data could provide deeper insight into medium repetitive sequences, occurrence of tandem repeats with hairpin like structures in *Silene* genomes [20] could be underestimated due to sequencing problems. Generally, it is known that sequencing of satellite repeats leads to underestimation of their abundance [37]. Furthermore, NGS methods could bias quantification of individual repetitive elements by occurrence of multiple identical reads [38] and lower efficiency in GC-rich templates sequencing [39].

By comparing the *S. vulgaris* and *S. latifolia* genomes we showed that the *gypsy* retroelement *Ogre* was the most divergent repetitive sequence, with almost no occurrence in the smaller genome of *S. vulgaris* and with a total count of about 25,000 in the *S. latifolia* genome (Table 1). Surprisingly, this element was not observed in the non-recombining part of the Y chromosome, suggesting that this element was not a major player in the evolution of large sex chromosomes in the dioecious *S. latifolia*.

The *Retand* retrotransposon was the most abundant repetitive DNA in *S. vulgaris* comprising a higher proportion of the *S. vulgaris* genome than in *S. latifolia*. Using BAC clones we have identified a full length *Retand* retroelement from the *S. vulgaris* genome and compared this sequence to the known *Retand* sequence from *S. latifolia* [26]. Our data clearly show that the gag and pol parts share about 80% similarity between the species, while the LTRs and ORF3, and ORF4 are much more divergent (Figure 2). Our findings are supported by the fact that, for comparison, we used a complex set of 454 data from *S. latifolia* [35]. High divergence of LTR sequences even in such closely related species could present a useful tool for studies of recent evolution of sex chromosomes via colonization by retrotrasposons. Especially dating and character of divergence between sex chromosomes can be precised by combination of gene based data with LTR evolutionary dynamics. The *Retand* element was found in the vicinity of the *SlAP3* gene, which could also suggest that gene rich regions co-localize with subtelomeres where *Retand* is a dominant element in both studied species. These data are in agreement with observation by Siroky *et al.* [40] showing, that euchromatin markers like early replication and H4 hyperacetylation can be detected at all subterminal chromosome regions.

*Copia* retrotransposons are the third most abundant elements in *S. latifolia* and the second more abundant in the *S. vulgaris* genome. The widespread presence of this element and its conserved pattern of distribution in these two species corresponds to observed data in other plant species [41].

The data presented here show that specific groups of repetitive elements have differentially proliferated in two closely related *Silene* species. Our data further suggest that transposable element expansion alone is not sufficient to explain genome size evolution and genome size differences between *S. latifolia* and *S. vulgaris*. Our data show that all of the repetitive elements observed in this study together cover ∼22% of *S. vulgaris* genome and ∼37% of entire genome in *S. latifolia* (Table 1). Which mechanisms of genome formation could explain this discrepancy? It has been shown that variation in intron length differs significantly between X and Y linked genes and their counterparts in autosomal loci in *S. vulgaris* [30]. This process along with other suggested mechanisms such as expansion/contraction of tandem repeats, illegitimate recombination [42], and different numbers of genes between species [43] are all candidates for explaining differences in genome formation and should be studied in further detail in these species.

What if the genome of *S. vulgaris* was reduced compared to *S. latifolia*? It has been shown that genomic DNA loss by unequal homologous recombination and illegitimate recombination of retrotransposons occurred in rice [44]. Evidence of a similar mechanism has been found in *Arabidopsis* species [43]. In both studies it was shown that the reduction partially affected all retroelements. This differs from our observation in *Silene* species that some retroelements display similar numbers between species (*Copia*, *Retand*) and some retroelements differ significantly (*Ogre*). These data suggest that genome size reduction has not been a key mechanism of genome size evolution in *S. vulgaris* and in *S. latifolia* since these species diverged from common ancestor.

### What is the chromosomal distribution of individual elements in related species?

Since the haploid chromosome number (*n*) is 12 in almost all *Silene* species, there is no evidence of large-scale reorganization or even polyploidization during the recent evolution of the genus. We expected that due to the stability of genomes in the *Silene* genus our data would only reveal patterns of expansion of individual repeats in various species. For our experiments, we selected related species with sex chromosomes (*S. dioica*) as well as two other species from *Caryophyllaceae* family, one with a small genome (*Dianthus caryophyllus*) and the other with a large genome (*Lychnis chalcedonica*) [32], [45]. Surprisingly, we found very distinct patterns of distribution of the *Retand* retroelement in these species. Although this element has strict subtelomeric organization in *S. latifolia*, *S. dioica* and *S. vulgaris*, its centromeric position in *Lychnis chalcedonica* reveals that large chromosomal rearrangements followed by fusion-fission events might have occurred during the evolution of *Caryophyllaceae* family (Figure 1). To confirm centromeric localization of *Retand* in *Lychnis chalcedonica* we performed bicolor FISH using both *Retand* and STAR-C probes (Figure S4). On the other hand, using telomeric probe for FISH experiments doesn't reveal occurrence of telomeric sequences in internal or centromeric parts of chromosomes (data not shown). Since there is limited amount of genetic and genomic data for *Lychnis chalcedonica*, further detailed study should be carried out to show cause of large chromosome evolution in this species.

*Ogre* retroelements are present only in dioecious species from the section *Melandrium*, in *S. latifolia* and in *S. dioica*. Surprisingly, this element was not found in the Y chromosomes. These data suggest that the expansion of this element is a very recent evolutionary event specific for the section *Melandrium*. It also suggests that the mode of retrotransposition could be connected with recombination machinery, which is not present in the non-recombining part of the Y chromosome.

The chromosomal distribution of *Copia* retroelements is conserved in all the species examined in this study. The ability of FISH probes to hybridize even on the chromosomes of more distinct species suggests low sequence divergence of the element during evolution. It further seems that *Copia* retroelements have been very stable in their numbers per genome during the long term evolution of the *Silene* species. The ratio of the total counts of *Copia* elements in *S. vulgaris* to *S. latifolia* is 2.7 (Table 1). This is almost exactly the same as the ratio between the genome sizes of these two species, suggesting that *Copia* retroelements keeps their copy numbers in the genome at least 7 Mya, which is the age of the oldest stratum in the sex chromosomes of *S. latifolia* [34]. The high sequence similarity of *Copia* retroelements could be a reason that these sequences are recognized *en bloc* by RNAi machinery and effectively silenced. On the other hand, the *Copia* retrotransposon is the most accumulated retroelement on the Y chromosome of *S. latifolia* [28]. This fact could suggest that there exist other mechanism(s) removing *Copia* elements apart from the Y chromosome in *S. latifolia* genome.

The tandem repeat STAR-C displayed a conserved centromeric pattern in all of the species from the *Caryophyllaceae* family. Along with TR1, these two tandem repeats are the most biased elements in *S. vulgaris* compared to *S. latifolia* in terms of total numbers. Since it is known that tandem repeats are amplified by non-equal crossing-over, replication slippage or via extrachromosomal circles [46], some of these mechanisms may differ between these two species. Unlike STAR-C, the distribution of TR1 and X.43.1 is conserved only in the genus *Silene* and *Lychnis*.

### Distribution of repetitive sequences in genic regions compared to the whole genome – a lesson from microsatellites

From the previous studies it is known that microsatellites are strongly accumulated on *S. latifolia* sex chromosomes [22]. Surprisingly, based on genomic library screenings [28], microsatellites are underrepresented in the genomic repetitive DNA pool. This could either be due to cloning problems with tandem-arrayed DNA or misinterpretation of FISH data. Although the FISH method is a robust methodical approach in terms of generating rough estimations of DNA element localization, it does not usually provide information about small-scale patterns of distribution of individual elements. To focus on the distribution and constitution of microsatellites within sex chromosomes, we analyzed six BAC clones, each of which contained a sex linked gene and/or its autosomal homologue (*Sl4*, *SlAP3*) previously isolated both from *S. latifolia* and *S. vulgaris* [30], [31]. Our data suggest that there are no significant differences in microsatellite numbers either between sex chromosome-linked loci or between corresponding regions in *S. latifolia* and *S. vulgaris* (Table S2). Even when we focused on specific microsatellites, which were shown to be overrepresented on *S. latifolia* Y chromosome (CA, GA, GC, CAA, CAG, GAA, TAA) (Table S3), we did not find any significant differences in satellite distribution. Surprisingly, and in contrast to data by Kubat *et al.*, some microsatellites (GA, GC, CAG, GAA, TAA) were more abundant in the X allele compared to the Y [22].

What happens if we compare the *SlAP3Y* locus to the *Sl4Y* locus in terms of microsatellite distribution? It has been estimated that the *Sl4Y* gene stopped recombining with its X linked counterpart about 7 Mya [34]. The *SlAP3* gene is situated in a region in which recombination was restricted between 1–2.5 Mya [31]. Total counts of all microsatellites in both regions based on Table S2 reveal no differences in the general representation of microsatellites in these regions. This would suggest that microsatellites do not contribute to Y chromosome formation significantly, at least in some genic regions. On the other hand, our recent experiments focused on sequencing the *S. latifolia* genome have revealed frequent long reads (454 based sequencing) composed of only a repetition of a specific satellite motif. Data in this study suggest that microsatellite accumulation has either local character or covers mainly non-genic regions.

## Materials and Methods

### Plant material and DNA isolation

Plants of *S. vulgaris* and of *S. latifolia* were planted in a cultivation room under standard conditions (t 24°C, 16 h light/8 h dark). Genomic DNA was isolated from young leaves using DNAeasy Plant Mini Kit (Qiagen).

### Construction and screening of genomic short insert plasmid and BAC libraries

*S. vulgaris* genomic DNA was processed by sonication into fragments with an average length of 600–1200 bp. Ends of fragments were treated with T4 DNA polymerase and further phosphorylated by T4 polynucleotide kinase. DNA fragments were ligated to the plasmid vector pSMART® LCAmp (Lucigen) using the Smart Cloning Kit (Lucigen) and transformed into *E. cloni* 10G competent cells. Clones were robotically picked with Genomic Solution G3 robot into 384 well plates, grown for 18 h, replicated, and frozen at −80°C. Clones were then grid in duplicate on Hybond N^+^ (Amersham, Biosciences) nitrocellulose membrane filters following a 4×4 pattern that allowed us to identify the well position and plate number of each clone. The filters were incubated and processed as described in [47]. The library contained 7,720 clones (603 bp average insert size) representing an equivalent of about 0.42% of the *S. vulgaris* genome. Screening was performed by radioactive hybridization with α^32^P and with the Prime-It II Random Primer Labelling Kit (Stratagene) according to the manufacturer's protocol. As a probe, the labeled *S. vulgaris* genomic DNA and amplified domains (with minor modifications according to [28]) *Ogre*, *Retand* and *Copia* reverse transcriptase, LINE endonuclease, and Au SINE. *Mutator*, *Mariner*, *hAT* and *CACTA* transposase gene and tandem repeats STAR-C, TR1, and X.43.1 were used. *S. latifolia* and *S. vulgaris* BAC libraries were screened according to Cegan *et al.* [31]. For BAC library screening probes derived from *Sl4* and *SlAP3* genes were used as described in Cegan *et al.* [31] and Marais *et al.* [30].

### DNA amplification

Positively hybridized clones were selected and used as a template for PCR with vector specific SL1 and SR2 primers. The PCR reaction profile included 25 cycles of 30 s at 94°C, 30 s at 60°C and 1 min at 72°C preceded by an initial denaturation (3 min at 95°C) and followed by a final extension step (5 min at 72°C). For the repetitive DNA elements, PCR amplification was carried out according to [28]. For the *Retand* reverse transcriptase amplification we used POL primers under conditions described in [26], for the *Ogre* reverse transcriptase we used primers C233-F 5′-CCCTTTACCGCCACTGACTA-3′ and C233-R 5′-TCAGTTGGGTCTAGGGTCGT-3′. Cycling conditions for Ogre RT included an initial denaturation of 2 min at 94°C, 35 cycles of 40 s at 94°C, 40 s at 55°C, 40 s at 72°C and a final elongation of 7 min at 72°C. PTC-200 (MJ Research) and T3000 (Biometra, Goettingen, Germany) thermal cyclers were used.

### Southern blot hybridization

Genomic DNA of *S. latifolia* (male and female) and of *S. vulgaris* were restricted by *Hind*III, *Msp*I, and *Hpa*II and then transferred by reverse Southern blotting onto Hybond N^+^ (Amersham, Biosciences) membrane filters. The *Ogre*, *Retand* and *Copia S. latifolia* reverse transcriptases were used as probes. Radioactive hybridization was performed as described in the construction and screening of the short insert library.

### Sequencing and bioinformatics analysis

Amplified PCR products were treated by ExoSAP, labelled by BigDye® Terminator Cycle Sequencing Kit according to the manufacturer's instructions and further purified by Agencourt® CleanSEQ® kit. Purified and labeled samples were sequenced (Sanger sequencing) with a 96 capillary sequencer ABI 3730xl according to the manufacturer's instructions. Sequences are available under GenBank accession numbers JN624389-JN624685.

BAC DNA was isolated and commercially sequenced from selected BAC clones using 454 sequencing with Roche GS FLX (GATC Biotech, Konstanz). 454 reads were assembled using MIRA3 [48], TGICL [49] and Roche GS De novo Assembler version 2.5.3 software. BAC sequencing and assembly statistics are described in Table S4. BAC sequences are available under GenBank accession numbers JQ289120–JQ289125. BAC contig annotations, based on BLAST with xml output and conversion for Geneious Pro software (Biomatters Ltd, Auckland, New Zealand; [50]), were made using TAIR9 cds and TAIR9 TE databases, the *S. vulgaris* transcriptome database (Taylor *et al.*, in preparation; http://silenegenomics.biology.virginia.edu/index.html). For graphic annotation in Geneious Pro sofware [50] we also used the vector and *E. coli* sequences, databases of short insert libraries of *S. latifolia* [28] and *S. vulgaris* (obtained in this paper), marker genes and gene prediction software Genscan [51].

Basic sequence analysis, sequence assembly and alignments were done with Geneious Pro software [50]. Multiple sequence comparisons were performed with MAFFT [52] (http://align.bmr.kyushu-u.ac.jp/mafft/online/server/) and BLAST online applications. A homology search was performed with BLAST [53]. Similarities with Repbase [54](http://www.giriinst.org/repbase/index.html) were found using CENSOR [55](http://www.giriinst.org/censor/index.php) and with Repeat Masker [56] (http://www.repeatmasker.org). For full length retroelement identification we used LTR Finder [57] (http://tlife.fudan.edu.cn/ltr_finder/) and JDotter [58] (http://athena.bioc.uvic.ca/QuickStart/JDotter). The other simple sequence analyses were completed using The Sequence Manipulation Suite - version 2 (SMS2) [59] (http://www.bioinformatics.org/sms2/). We used Bio-Linux 6.0 operating system [60].

### Data analysis

Nucleotide alignment visualization of full length elements from BACs was done using Lalnview [61]. The comparative analysis of the *Retand* full length elements from BAC clones to genomic 454 reads from *S. latifolia* (male and female) was done on PROFREP server (beta version) (the server is maintained by the Laboratory of Molecular Cytogenetics, Institute of Plant Molecular Biology, Ceske Budejovice, Czech Republic) with E-value cutoff of 10^−15^.

To identify microsatellites, we used Perfect Microsatellite Repeat Finder (http://sgdp.iop.kcl.ac.uk/nikammar/repeatfinder.html) on each BAC sequence, with the default parameters (minimum number of repeats = 3, minimum repeat unit length = 2 and maximum repeat unit length = 100).

To calculate a number of sequences per genome, we took the actual number of each type of element in the library and multiplied it by genome coverage of the library. The % of the genome was calculated by multiplication of number of sequences per genome by average element size (kb) (Table 1, Table S1).

### Fluorescence *in situ* hybridization on metaphase chromosomes

Slides with mitotic metaphase chromosomes of *S. latifolia*, *S. vulgaris*, *S. dioica*, *Lychnis chalcedonica* and *Dianthus caryophyllus* were treated as described in [62] with slight modifications. Slide denaturation was performed in 7∶3 (v/v) formamide: 2× SSC for 2 min at 72°C. Slides were immediately dehydrated through 50%, 70%, and 100% ethanol (−20°C), and air dried. The probe was denatured at 70°C for 10 min, and 100 ng of the denatured probe was added at room temperature and hybridized for 18 h at 37°C. Slides were analyzed using an Olympus Provis microscope, and image analysis was performed using ISIS software (Metasystems). DNA was labeled with Fluorolink Cy3-dUTP (Amersham Pharmacia Biotech) (red labeling) and Spectrum Green (Vysis) (green labeling) in combination with the nick translation mix (Roche).

The probe for STAR-C was synthesized by VBC-Genomics (Vienna) with Cy3 modification on the 5′end. Chromosomes were stained with DAPI (4′ 6′- diamidino-2-phenylindole).

## Supporting Information

Figure S1 Southern blot analysis. Male (M) and female (F) genomic DNA of *S. latifolia* and *S. vulgaris* (SV) was restricted using *Hind*III, *Msp*I and *Hpa*II. Hybridization was carried out with reverse transcriptase of *Ogre*, *Retand* and *Copia* retroelements as probes. The 1 kb DNA ladder (L 1 kb) is indicated.
(TIF)

Figure S2 Chromosomal distribution of 25S rDNA (A–E) and tandem repeats STAR-C (F–J), TR1 (K–O) and X.43.1 (P–T) in five species from the *Caryophyllaceae* family as determined by FISH. Mitotic metaphase chromosomes of *S. latifolia* (A, F, K, P), *S. dioica* (B, G, L, Q), *S. chalcedonica* (C, H, M, R), *S. vulgaris* (D, I, N, S) and *Dianthus caryophyllus* (E, J, O, T) were counterstained with DAPI (blue). The probes were labeled with Cy3-conjugated nucleotides (red). The X and Y chromosomes are indicated, bars indicate 10 µm.
(TIF)

Figure S3 Comparison of STAR-C tandem repeat signal intensities in *S. latifolia* (A–C) and *S. vulgaris* (D–F) by FISH. Metaphase chromosomes were counterstained with DAPI (blue); the STAR-C probe was labeled with Cy3-conjugated nucleotides (red). Exposition time is indicated in the figure. The X and Y chromosomes are indicated, bar represents 10 µm.
(TIF)

Figure S4 Chromosomal distribution of STAR-C (red) and *Retand* (green) on *L. chalcedonica*. Mitotic metaphase chromosomes were counterstained with DAPI (blue).
(TIF)

Table S1 Proportions of individual elements in the genomes of *S. latifolia* and S. vulgaris (* data from Cermak et al. [*28*] *).*
(PDF)

Table S2 Percentage of microsatellites in genomic loci containing the *SlAP3* and *Sl4* genes in *S. latifolia* and *S. vulgaris*. Di-, tri-, and tetranucleotide microsatellites were calculated separately.
(PDF)

Table S3 Percentage of specific microsatellites in BAC clones containing the *SlAP3* and *Sl4* genes in *S. latifolia* and *S. vulgaris*. Satellite units were selected based on data by Kubat *et al.* [20] showing accumulation of several microsatellites on Y chromosome of *S. latifolia*.
(PDF)

Table S4 BAC sequencing and assembly statistics.
(PDF)
