# Supplementary figures and images for: Genomic Diversity in Two Related Plant Species with and without Sex Chromosomes - Silene latifolia and S. vulgaris
Source: PLoS One. 2012 Feb 29;7(2):e31898. doi: 10.1371/journal.pone.0031898 (PMC3290532; doi:10.1371/journal.pone.0031898)

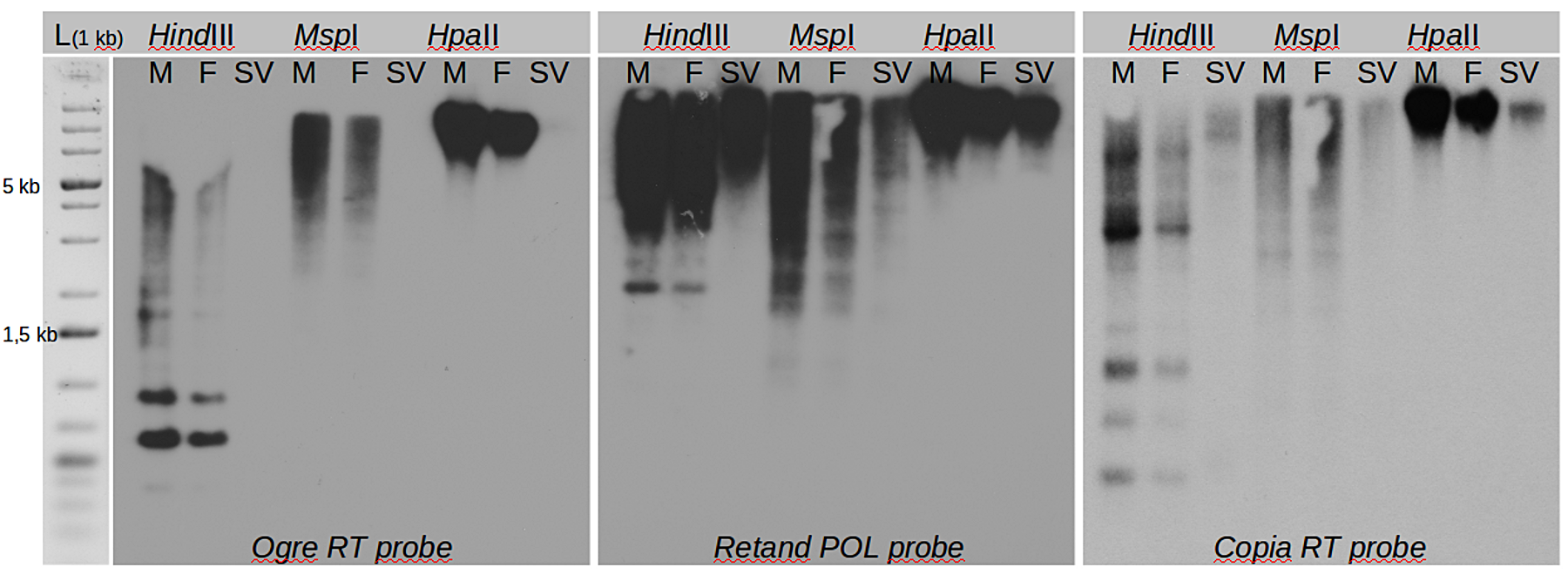

Supplement: Figure S1 — Southern blot analysis. Male (M) and female (F) genomic DNA of S. latifolia and S. vulgaris (SV) was restricted using HindIII, MspI and HpaII. Hybridization was carried out with reverse transcriptase of Ogre, Retand and Copia retroelements as probes. The 1 kb DNA ladder (L 1 kb) is indicated. (TIF) [file pone.0031898.s001.tif]

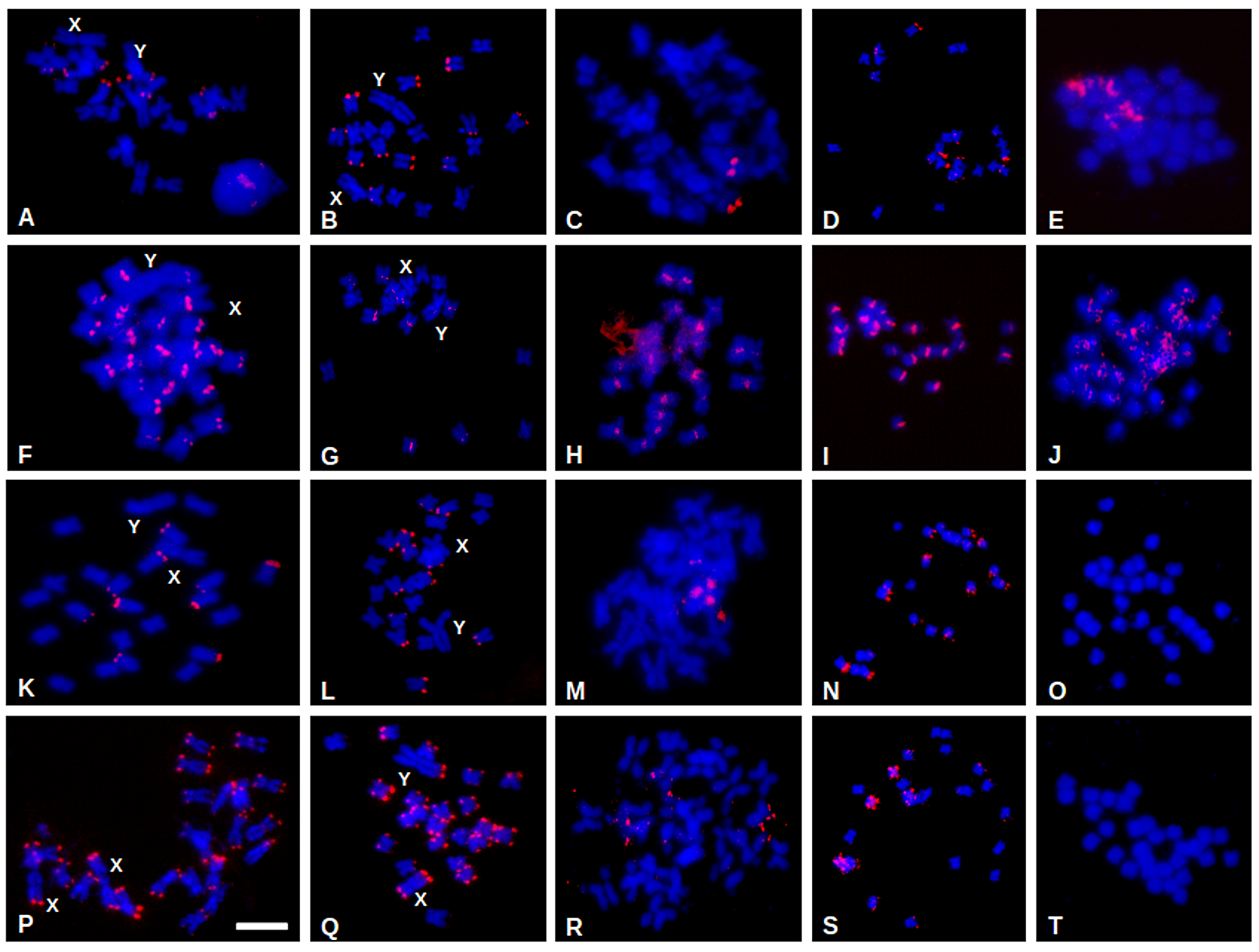

Supplement: Figure S2 — Chromosomal distribution of 25S rDNA (A–E) and tandem repeats STAR-C (F–J), TR1 (K–O) and X.43.1 (P–T) in five species from the Caryophyllaceae family as determined by FISH. Mitotic metaphase chromosomes of S. latifolia (A, F, K, P), S. dioica (B, G, L, Q), S. chalcedonica (C, H, M, R), S. vulgaris (D, I, N, S) and Dianthus caryophyllus (E, J, O, T) were counterstained with DAPI (blue). The probes were labeled with Cy3-conjugated nucleotides (red). The X and Y chromosomes are indicated, bars indicate 10 µm. (TIF) [file pone.0031898.s002.tif]

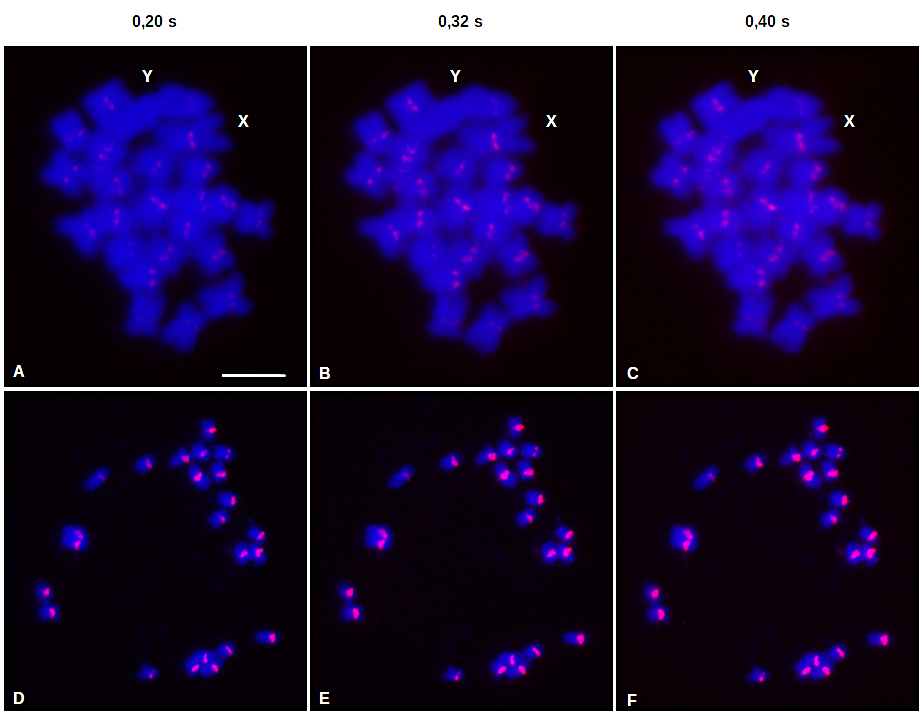

Supplement: Figure S3 — Comparison of STAR-C tandem repeat signal intensities in S. latifolia (A–C) and S. vulgaris (D–F) by FISH. Metaphase chromosomes were counterstained with DAPI (blue); the STAR-C probe was labeled with Cy3-conjugated nucleotides (red). Exposition time is indicated in the figure. The X and Y chromosomes are indicated, bar represents 10 µm. (TIF) [file pone.0031898.s003.tif]

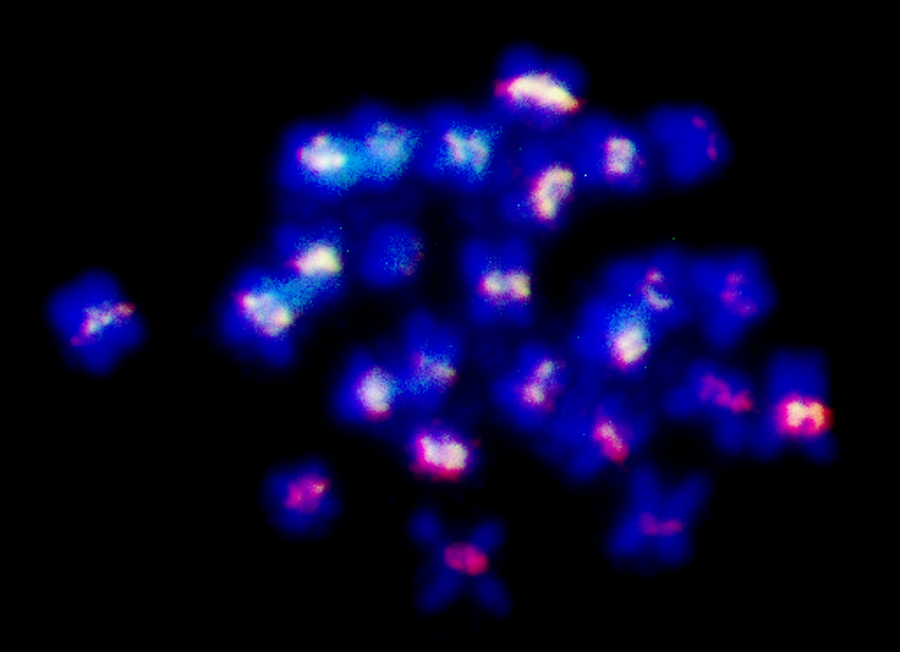

Supplement: Figure S4 — Chromosomal distribution of STAR-C (red) and Retand (green) on L. chalcedonica. Mitotic metaphase chromosomes were counterstained with DAPI (blue). (TIF) [file pone.0031898.s004.tif]
