# Supplementary material for: Genomic Diversity in Two Related Plant Species with and without Sex Chromosomes - Silene latifolia and S. vulgaris
Source: PLoS One. 2012 Feb 29;7(2):e31898. doi: 10.1371/journal.pone.0031898 (PMC3290532; doi:10.1371/journal.pone.0031898)
Supplement: Table S1 — Proportions of individual elements in the genomes of S. latifolia and S. vulgaris (* data from Cermak et al. [28] ). (PDF) [file pone.0031898.s005.pdf]

Table S1. Proportions of individual elements in the genomes of *S. latifolia* and *S. vulgaris (* data from Cermak et al. [28]).*

Type of element	Total number of clones containing respective elements										
	Silene vulgaris						*Silene latifolia **				
	**Number of clones**	**Proportion of clones (%)**	**Element size (kb)**	**Number of clones per genome**	**kb**	**% of genome**	**Number of clones**	**Proportion of clones (%)**	**Number of clones per genome**	**kb**	**% of genome**
**Retroelements**	127	41				22.9%	232	62,3			36.4%
**LTR**	125	40,4				22.8%	223	59,9			36.1%
**Gypsy**	**58**	**18,8**				**12.7%**	**179**	**48,1**			**32.5%**
Athila	3	1	7	714	4998	0.5%	32	8,6	10000	70000	2.7%
Ogre	0	0	24	0	0	0	80	21,5	25000	600000	22.7%
Peabody	8	2,6	6,5	1904	12376	1.1%	36	9,7	11250	73125	2.8%
Retand	47	15,2	11	11190	123090	11.2%	31	8,3	9687	106562	4%
**Copia**	67	21,6	7	15952	111664	**10.1%**	44	11,8	13750	96250	3.6%
**Non - LTR**	**2**	**0,6**				**0.1%**	**9**	**2,4**			**0.3%**
SINE	0	0	0,5	0	0	0	0	0	0	0	0
LINE	2	0,6	2,5	476	1190	0.1%	9	2,4	2812	7031	0.3%
**DNA transposons**	**6**	**1,9**	**4**	**1428**	**5117**	**0.6%**	**3**	**0,8**	**938**	**4532**	**0.2**
DNA/EnSpm	2		6	476	2856	0.3%	2		625	3750	0.1%
DNA/hAT	1		3	238	714	0.1%	0		0	0	0
DNA/MuDR	1		2,5	238	595	0.1%	1		313	782	0.1%
DNA/P	2		2	476	952	0.1%	0		0	0	0
**Tandem repeats**	**177**	**57,1**				**0.3%**	**137**	**36,9**			**0.6%**
Tandem repeat STAR	118	38,1	0,043	28095	1208	0.1%	29	7,8	9063	389	0.02%
Tandem repeat X.43.1	19	6	0,312	4523	1411	0.12%	101	27,2	31563	9847	0.37%
TR1	40	12,9	0,074	9523	704	0.06%	7	1,9	2188	161	0.01%
**All Repetitive elements**	**310**	**100**				**23.4%**	**372**	**100**			**36.8%**
