# Supplementary material for: Genomic Diversity in Two Related Plant Species with and without Sex Chromosomes - Silene latifolia and S. vulgaris
Source: PLoS One. 2012 Feb 29;7(2):e31898. doi: 10.1371/journal.pone.0031898 (PMC3290532; doi:10.1371/journal.pone.0031898)
Supplement: Table S2 — Percentage of microsatellites in genomic loci containing the SlAP3 and Sl4 genes in S. latifolia and S. vulgaris. Di-, tri-, and tetranucleotide microsatellites were calculated separately. (PDF) [file pone.0031898.s006.pdf]

Table S2. Percentage of microsatellites in genomic loci containing the *SlAP3* and *Sl4* genes in *S. latifolia* and *S. vulgaris.* Di-, tri-, and tetranucleotide microsatellites were calculated separately.

Dinucleotides

Length	SlAP3Y	SlAP3X	SvAP3	SlY4	SlX4	Sv4
6	17.255	16.280	19.870	15.163	16.019	16.952
7	5.464	4.659	5.195	5.501	6.786	6.983
8	1.245	0.786	2.279	1.552	1.112	1.445
9	0.484	0.393	1.094	0.635	0.890	1.059
10	0.104	0.000	0.091	0.212	0.556	0.144
11	0.173	0.056	0.182	0.000	0.111	0.241
12	0.104	0.000	0.091	0.071	0.000	0.096
13	0.035	0.056	0.000	0.000	0.000	0.048
14	0.035	0.056	0.091	0.000	0.000	0.000
15	0.069	0.000	0.000	0.141	0.000	0.000
16	0.000	0.000	0.000	0.000	0.000	0.000
17	0.000	0.000	0.000	0.000	0.000	0.000
>18	0.104	0.056	0.091	0.282	0.111	0.144

Trinucleotides

Length	SlAP3Y	SlAP3X	SvAP3	SlY4	SlX4	Sv4
9	3.769	3.368	4.375	3.315	3.115	1.4.671
10	1.625	1.572	2.005	0.987	1.112	1.397
11	0.865	0.618	0.456	0.635	0.111	0.867
12	0.415	0.225	0.456	0.141	0.111	0.289
13	0.035	0.393	0.182	0.282	0.000	0.144
14	0.138	0.112	0.365	0.071	0.111	0.385
15	0.000	0.168	0.091	0.071	0.111	0.193
16	0.000	0.000	0.000	0.071	0.000	0.048
17	0.035	0.000	0.091	0.071	0.222	0.096
18	0.000	0.056	0.091	0.071	0.000	0.048
19	0.000	0.000	0.000	0.000	0.222	0.048
20	0.035	0.000	0.000	0.000	0.111	0.000
>20	0.138	0.112	0.000	0.564	0.222	0.096

Tetranucleotides

Length	SlAP3Y	SlAP3X	SvAP3	SlY4	SlX4	Sv4
12	0.346	0.281	0.456	0.353	0.222	0.674
13	0.173	0.337	0.182	0.212	0.222	0.241
14	0.035	0.056	0.000	0.000	0.111	0.048
15	0.000	0.000	0.091	0.071	0.000	0.000
16	0.035	0.000	0.182	0.000	0.000	0.000
17	0.000	0.000	0.000	0.071	0.222	0.048
18	0.000	0.056	0.000	0.000	0.000	0.048
19	0.035	0.000	0.000	0.000	0.000	0.000
20	0.035	0.000	0.091	0.000	0.000	0.000
>20	0.035	0.000	0.000	0.000	0.000	0.048
