# Supplementary material for: Genomic Diversity in Two Related Plant Species with and without Sex Chromosomes - Silene latifolia and S. vulgaris
Source: PLoS One. 2012 Feb 29;7(2):e31898. doi: 10.1371/journal.pone.0031898 (PMC3290532; doi:10.1371/journal.pone.0031898)
Supplement: Table S3 — Percentage of specific microsatellites in BAC clones containing the SlAP3 and Sl4 genes in S. latifolia and S. vulgaris. Satellite units were selected based on data by Kubat et al. [20] showing accumulation of several microsatellites on Y chromosome of S. latifolia. (PDF) [file pone.0031898.s007.pdf]

Table S3. Percentage of specific microsatellites in BAC clones containing the *SlAP3* and *Sl4* genes in *S. latifolia* and *S. vulgaris*. Satellite units were selected based on data by Kubat *et al.* [20] showing accumulation of several microsatellites on Y chromosome of *S. latifolia*.

Dinucleotide

Unit	SlAP3Y	SlAP3X	SvAP3	SlY4	SlX4	Sv4
CA	3.84	2.41	3.65	4.30	3.67	3.56
GA	4.25	4.49	4.74	3.31	4.34	4.67
GC	0.24	0.56	0.27	0.56	0.00	0.14

Trinucleotide

Unit	SlAP3Y	SlAP3X	SvAP3	SlY4	SlX4	Sv4
CAA	0.55	0.51	0.36	0.35	0.22	0.43
CAG	0.17	0.28	0.27	0.07	0.11	0.05
GAA	0.28	0.34	0.27	0.85	0.33	0.34
TAA	0.41	0.67	1.09	0.71	0.78	0.77
