# Supplementary material for: Genomic Diversity in Two Related Plant Species with and without Sex Chromosomes - Silene latifolia and S. vulgaris
Source: PLoS One. 2012 Feb 29;7(2):e31898. doi: 10.1371/journal.pone.0031898 (PMC3290532; doi:10.1371/journal.pone.0031898)
Supplement: Table S4 — BAC sequencing and assembly statistics. (PDF) [file pone.0031898.s008.pdf]

Table S4 . BAC sequencing and assembly statistics.

BAC Name	Gene name	Number of Reads	Number of Bases	Number of Assembled Reads	Large Contig/Scaffold Number (>1 kb)	Average Large Contig/Scaffold Size	Percent Assembled (%)
**65P13**	**SlX4**	200537	38443944	170587	3	22463	0.85
**93L17**	**SlY4**	1154	1575745	932	23	6222	0.81
**62M2**	**Sv4**	69648	16725302	63674	30	6921	0.91
**251L13**	**SlAP3X**	6275	2056538	5797	56	3180	0.92
**30L22**	**SlAP3Y**	82974	20293397	72221	39	7023	0.87
**91M20**	**SvAP3**	23398	5800501	21206	6	18284	0.91
